# Integrated care in practice: lessons from three tiers of healthcare provider and commissioner staff in two London Integrated Care Systems

**DOI:** 10.1192/bjo.2025.10841

**Published:** 2025-09-15

**Authors:** Derek K. Tracy, Lisa C. Lloyd, Sukhwinder S. Shergill, Kara Hanson

**Affiliations:** South London NHS Foundation Trust, London, UK; The Institute of Psychiatry, Psychology and Neuroscience, King’s College London, UK; Brunel Medical School, London, UK; Coventry and Warwickshire Partnership NHS Trust, Coventry, UK; Kent and Medway Medical School, Canterbury, UK; Faculty of Public Health and Policy, London School of Hygiene and Tropical Medicine, UK

**Keywords:** Integration, integrated care, social care, healthcare, NHS 10-Year Plan

## Abstract

**Background:**

To better meet the growing demand and complexity of clinical need, there is a broad international trend towards greater integration of various elements of health- and social care. However, there has been a lack of research aimed at understanding how healthcare providers have experienced these changes, including facilitators and inhibitors of integration.

**Aims:**

This study set out to generate new understandings of this from three UK staffing ‘levels’: ‘micro’ frontline workers, a ‘meso’ level of those leading a healthcare organisation and a ‘macro’ level of commissioners.

**Method:**

Using Rogers’ Diffusion of Innovation framework, qualitative analysis of individual interviews from provider staff perceptions was undertaken at these three levels (total *N* = 33) in London.

**Results:**

English legislation and policy captured the need for change, but fail to describe problems or concerns of staff. There is little guidance that might facilitate learning. Staff identity, effective leadership and culture were considered critical in implementing effective integration, yet are often forgotten or ignored, compounded by an overall lack of organisational communication and learning. Cultural gains from integration with social care have largely been overlooked, but show promising opportunities in enhancing care delivery and experience.

**Conclusions:**

Findings are mixed insofar as staff generally support the drivers for greater integration, but their concerns, and means for measuring change, have largely been ignored, limiting learning and optimisation of implementation. There is a need to emphasise the importance of culture and leadership in integrated care, and the benefits from closer working with social care.

There is a widespread international move to more integrated health- and social care systems. The drivers for this are unambiguous.^1^ Populations are ageing and becoming more clinically complex, and siloed specialist service provision is often poorly structured to meet this multimorbidity. In parallel, there is a widely acknowledged workforce recruitment and retention crisis in these sectors,^1^ and growth in funding alone seems unlikely to occur in a manner adequate to match this need.^2^

In England, as part of this pattern, health- and social care^a^ have been undergoing arguably their most profound reformation of the past 50 years.^3^ Policy and legislation, notably first the 2019 National Health Service (NHS) Long-Term Plan (LTP)^4^ and 2022 Health and Care Act^5^ and, most recently, the 2025 10-Year Health Plan for England^6^ (commonly referred to as ‘the 10-Year Plan’) have brought them ever closer together into ‘Integrated Care Systems’ (ICSs) under the authority of a managing Integrated Care Board (ICB). These structures are complex, although broadly echo those of other high-income countries:^7^ Fig. 1 gives an overview of these and their accountability in England, taking recent legislative changes into account, with Fig. 2 illustrating the roles and interfaces of social care delivered via local authorities.

**Fig. 1:**
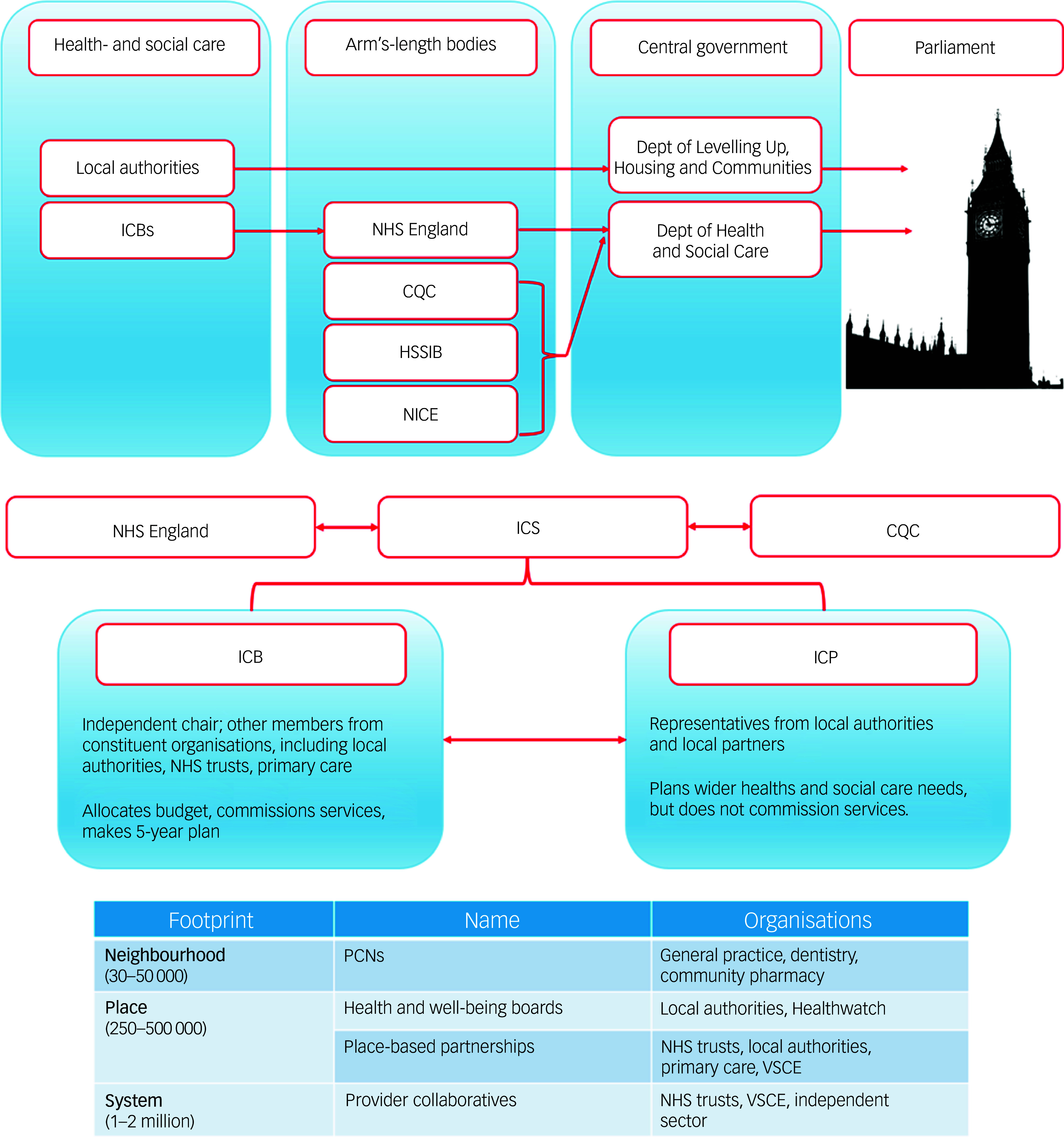
Accountability within the health- and care system in England is complex. The top half of the figure shows this following the 2022 Health and Care Act (adapted from data from the King’s Fund^24^). Of note, the 2025 National Health Service (NHS) 10-Year Plan^6^ does not fundamentally change this. Arm’s-length bodies are executive and non-departmental public bodies that support the work of government departments. CQC, Care Quality Commission; HSSIB, Healthcare Services Safety Investigation Branch; NICE, National Institute for Health and Care Excellence. Note that, since this work commenced, further legislative changes mean that the National Health Service (NHS) in England will be absorbed into the Department of Health and Social care by 2027, although the principles of the described relationships will remain.^25^ The bottom half of the figure describes the relationship between Integrated Care Systems (ICSs), Integrated Care Boards (ICBs), NHS England and other partners under the 2022 Health and Care Act. ICB sizes and functions have also retracted following the aforementioned changes, although again, the principles of their operations remain. Note that many organisations, such as individual NHS trusts, might work across more than one geographical ‘level’ in this figure, and there will be variation between ICSs/ICBs in their underlying detail. ICPs, Integrated Care Partnerships; PCNs, Primary Care Networks; VSCE, voluntary, community and social enterprise (adapted from data from the King’s Fund^26^).

**Fig. 2:**
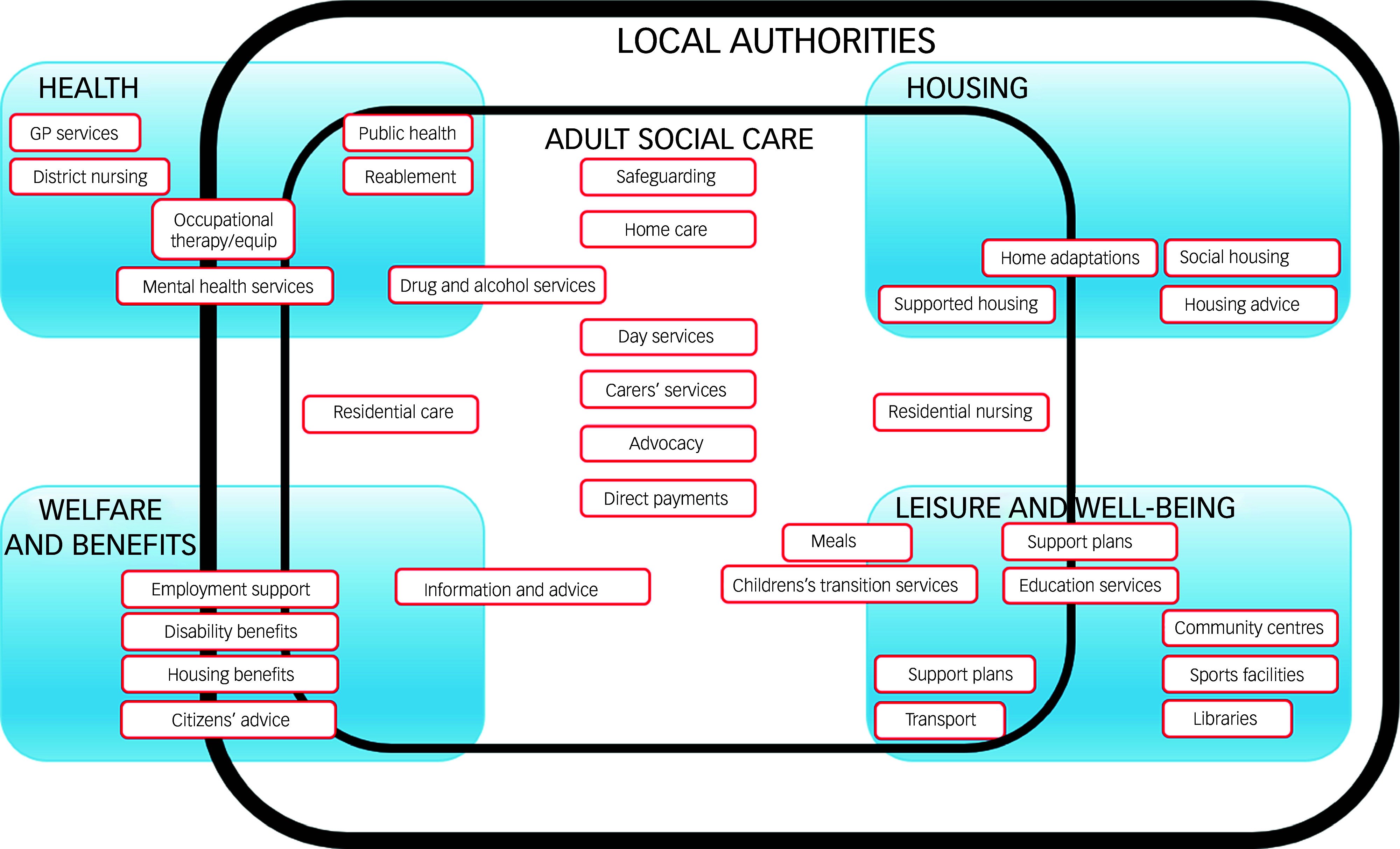
Schematic illustration of the many roles and complex interfaces of local authorities and adult social care (figure based on, and adapted from, data from the National Audit Office^27^).

To date there is relatively limited empirical evidence on how these changes have been experienced by those whose work is being integrated.^8^ Some research has looked at specific staff groups, including district nurses and social worker ‘case managers’ in the Netherlands,^9^ nurse navigators in Australia^10^ and care coordinators in in France.^11^ Other work has explored the impact of integration on specific pathways, including dementia,^12,13^ Parkinson’s disease,^14^ frailty,^15,16^ oncology,^17^ liver disease,^18^ polycystic ovary disease^19^ and neonatal services.^20^ Common themes that emerged included that staff were positive about more ‘joined-up care’, but emphasised the need for more active communication on changes, often feeling disconnected from implementing leaders. In terms of broader service structural changes, Round et al^21^ explored effects on hospital admissions and nursing home placements. Key lessons included the importance of strong clinical leadership, shared ownership and inbuilt evaluation. McDermott et al^22^ looked at primary care in the UK at the time of its move to clinical commissioning groups (CCGs) from primary commissioning groups (PCTs), describing a disconnect between strategic plans and initiatives that focused on incentivising and supporting sustainable practice. More recently, Mitchell et al^23^ reported on the perspectives of GPs in primary care services about pending integrating changes in England.

## Aims and objectives

The aim of this paper is to generate new understandings of the implementation of large-scale integrated care systems, taking the specifics of English mental and community physical healthcare organisations and ICSs in practice, with the objectives of analysing early response to these changes across different staff levels to understand the perceived appropriateness and effectiveness of various elements of policy shift, and to identify factors that facilitate and inhibit more integrated care.

## Method

Hughes et al^28^ wrote of the need to use a strong structuration theory to explain integrated care, noting the specifics of changes in English systems. In line with their work and recommendations, we studied the implementation of integrated care at three different scales – the micro level (frontline teams), the meso level (the leadership of an NHS Trust) and the macro level (the ICB itself).

The ambition of changes suggests the need for a broad framework that can incorporate a wide variety of opinions across a range of different service types. Rogers’ ‘Diffusion of Innovation’^29^ has been widely applied in healthcare. A strength of Rogers is that its breadth and lack of specificity, and flexibility without being overly prescriptive in application, allow it to be utilised as a deductive scaffolding framework upon which data can be applied in a very wide number of areas in health. These factors led to it being chosen for this study, where we used its seven over-arching elements (see Table 1) for that deductive framework upon which we could explore how a service delivery innovation diffused in practice.

**Table 1:** The overarching themes, main themes, subthemes and key example quotations from the qualitative interviews

Overarching theme	Main themes	Subthemes	Key examples from interviews
Prior conditions	Social norms		‘I think in theory [to] bring them all together working collaboratively … would be an excellent thing to do, a rationale for the right thing to do in services’ (G1P8)
	Individuals’ interests		‘life is complex … people live longer … often have more illnesses or healthcare problems, so they need more than one service. Or team or professional … not very good at moving people between it. The models are for the team, not for the person’ (G1P6)
	Economic interests		‘the cynic in me says there was nobody in government that said, this [the existing clinical model] is all plainly wrong … it’s more the backwash from austerity … trying to keep people out of hospital’ (G2P12)
Innovation characteristics	Relative advantage	Quality of care	‘it’s what I would want for myself, for my family’ (G1P13).
		Equality of care and preventative care	‘getting communities much more involved in shaping what we provide … a genuine partnership between our professionals, our clinicians, our experts in social care and our local populations’ (G2P5)
		Patient and carer experience	‘actually having it in just one place … they all communicate with each other … So my social worker knows what my nurse is doing … My nurse knows what my social worker is doing’ (G1P2)
		Workflow and primary care interface	‘we could be helping GPs a lot more with the with the day-to-day stuff’ (G2P10)
		Workload	‘Why have we got five home visits from nurses? A district nurse, a mental health nurse or social worker, a care worker when you can reduce that to one or two and make every contact count’ (G2P6)
		A learning system	‘I’m excited about working with the bigger group of professionals of learning from them of thinking in different ways … it’s not just that other professionals treat their bit of the person, but they add something else to the mix as well, which is rich’ (G1P13)
		Finances	‘We have to make the best use of the resources … we self-evidently don’t do that currently … Demand will be infinite. Capacity will be limited’ (G3P2)
		Leaner and more efficient commissioning	‘whilst we might be excited that a particular practitioner delivers outstanding results in a particular set of conditions, what we want is outstanding results for the whole population … innovation is good, but it can only be good if it can be translated at scale. It’s actually a problem that we have a celebration of innovation, an innovation-itis in the NHS … we find one thing that works somewhere … it’s never properly independently evaluated … what are the barriers that stop this being launched at scale … to get the benefits for a population as opposed to the lucky few … and it dies off’ (G3P2)
		Estates, back-office, information technology	‘less duplication, less paperwork … particularly the rather dreary, dull stuff, and allow people to spend more time with patients’ (G2P8)
		Social care ‘in the mix’	‘this is the latest vehicle to try [more integration] … and really … the first [to try] address the real elephant in the room … without good interdigitation with social care, it won’t succeed’ (G3P4)
	Compatibility with values and experiences	More equitable community care	‘our goal, particularly for me … [to] support the more diverse and the more disadvantaged populations’ (G2P11)
		‘Saving money’	‘If you got at the moment me doing one job and someone else doing different jobs and you can amalgamate us, simple maths says it’ll be cheaper’ (G1P4) ‘I do find it a bit odd, but also a bit disappointing that somehow saving money is seen as a bad thing. Where it actually ought to be a fantastic thing so we can deliver a particular innovation, intervention in healthcare for 20% less cost. That means we can do 20% more of those interventions. What’s not to like?’ (G3P5)
		Deskilling	‘I’m a trained physiotherapist … I’m hardly teaching a nurse to become a physio, and I don’t think they’d want that anyway. I don’t want to be a nurse. No disrespect … will we be able to keep up our specialist skills?’ (G1P11) ‘you actually become more aware of what your expertise is in an integrated system with lots of professionals … what bits you do bring … there’s an expertise in being able to think in an integrated way … which I think we need more of … the future skill isn’t specialist knowledge you can Google or a machine can tell you, it’s the ability to integrate all that information into a living person in front of you and their life and their care’ (G2P10)
		Risk and stigma	‘we aren’t able to access things across trusts and therefore they’re [potential] causes of a huge amount of clinical errors and serious incidents’ (G2P10)
	Complexity in use, understanding	Focusing on the ‘wrong minority’	‘Are the needs of the wider range of more general patients being sacrificed…someone who is referred with ‘just’ depression? This [model] all assumes everyone needs lots of professionals’ (G1P10)
		Work on the ‘wrong’ patients	‘one could … argue that almost everyone should come to us. I worry that … It’s about inappropriately lower referral thresholds. We will be asked to see people outside our commissioned base. We will be seeing less severe instances or cases of ill health’(G1P10)
		Governance and disputes	‘currently the legal form is organisational [NHS trusts] … [which] have the ultimate legal accountability. So the ability of the ICB to influence the system when individual organisations don’t want to [engage] … those governance issues haven’t really been ironed out’ (G2P13)
	Trialability and reversibility before committing		
	Observability of results		‘Patients and carers will tell you the system is easier to navigate, that they feel better looked after that their healthcare conditions are improved and they are dealt with in a more holistic way with staff who are interested in them as whole people rather than just “illness parts”’ (G1P14)
Stage of innovation	Knowledge, awareness		‘I think those things are only meaningful if you’re at a certain level within the organisation and the further away you get away from direct patient care, the less relevant or meaningful it becomes’ (G2P13)
	Persuasion	Relative position	‘we have to do things differently. We can’t keep going as we are. We don’t have enough staff as it is … to try work better across traditional boundaries or teams or services’ (G1P1) ‘We are ahead of the curve here in Bexley … it’s a good thing, I guess, in principle, but it’s causing us problems. We don’t know what we’re doing … not clear to me that we have any template to map against … I think we are probably more advanced in having social care as part of this’ (G1P1}
	Decision to adopt or reject		‘It’s not that staff are unable to see the benefits … when people are feeling incredibly pressured … [why] make me do this’ (G1P5)
	Implementation of innovation	Care quality, workflow, learning	‘it’s quite nice … continual conversation about changes … new services, ways of working … makes people more … efficient … it’s just nice to be personal relationship with … different professionals that work in different ways’ (G1P) ‘Good work on joint training … looking at GPs’ work, working with mental practitioners in primary care settings … learning … the results show it can help … for both the GP and the mental health professionals’ (G1P7)
		Contractual variation, duplication	‘the admin gets … £3000 less if there you have a health contract … hundreds of things that actually that come up [via] workforce … we [health staff] get birthday leave and they don’t … the social workers [were] invited to our annual celebration dinner but they had to pay for their tickets … all gets in the way and actually for frontline staff that’s really important’ (G2P5)
		Financial behaviours	‘as a group of finance directors within NW London [we] spend less time arguing … more time collaborating’ (G2P12)
		Early working with the local authority	‘We can’t get anywhere actually, (a) because of social care, (b) because of housing and education … working with schools and (c) dealing with children, adolescents … at an early stage’ (G2P1)
	Confirmation		
Adopters’ characteristics	Identity		‘It’s that poxy purple lanyard that says “Bexleycare”. No one outside knows what it means. You do a home visit and people think you’re from a care agency or something … NHS people are proud of the NHS. They want to be part of that, not “Bexleycare”’ (G1P2)
	Professional role and growth		‘So far my feeling has been … actually quite an eye opener in terms of the difference between the local authority and mental health … I was watching our [physical] health colleagues … be much better at … collecting and auditing checking … but they are [also] much better than us [mental health] following latest NICE guidelines latest protocols etc and having strategies in place’ (G1P5) ‘confusing … [there’s a] term, a “practitioner” where people fit certain criteria but it doesn’t matter their profession … where as a professional do I fit in this?’ (G1P7)
	Leadership		‘Leaders need to watch and cultivate the spaces … help deliver that with our staff’ (G3P1)
	Culture more broadly		‘To be frank I think health have always been smarter. Politicians listen to the NHS. But then … you guys [health] give them evidence, you lobby, you’re effective. We [social care] moan about it, but we don’t do the research. You all publish stuff, we’re reading textbooks from the 60s and then complaining no one is listening. If you want to get a social worker to read what you’re thinking, you need to put it in the *Guardian* [newspaper] not a science journal. We need to be better … or smarter. We need to copy … well, learn this from health’ (G1P4) ‘People gain through being present with others rather than being told well do it this way … culture we absorb by being in each other’s presence … an osmosis of some kind’ (G1P5)
Communication behaviour			‘I think we have been updated, but I don’t feel listened to … It hasn’t been a two way … We know enough, we just don’t feel listened to … it does your head in’ (G1P4) ‘come see us, talk to us, listen to us … it felt emotionally like staff were screaming and shouting and trying to be heard … Managers always underestimate the value of their presence on the floor … Ask how best to spend this money, talk to us! Don’t make the decisions without that’ (G1P4)
Time to adaptation			‘I’m not sure that I think ICSs will exist in three years’ time … a new political party will come in. They’ll have had a brighter idea and it will be reorganised … we are changing every three, five years’ (G2P13)
The surrounding social system	Internal relationships		‘what most reorganisations do is break them down … you have to spend the next two or three years building them back up’ (G2P9)
	External relationships	ICS relationships	‘Like two parents who already have children deciding they want to marry … and don’t introduce the kids till after you’re married. It just doesn’t work. They could have done that bit better’ (G1P2)
		The local authority	‘The hearts and minds of our social care and local authority colleagues are … one of the biggest weaknesses, which is relationship, cultural, attitudinal, perspectives … just different from ours … difficulties we have partly come from [this]’ (G3P2)
		Local and national government, NHS England	‘A local authority councillor comes in and says I’ve no idea what you’re talking about, I don’t like that and here are our demands … never seen such demands before … is it you asking to discuss something or are you just talking at each other in different languages from positions that are irreconcilable … extremely difficult’ (G3P5)

NHS, National Health Service; ICB, Integrated Care Board; ICS, Integrated Care System; NICE, National Institute for Health and Care Excellence.

### Study settings

**Fig. 3:**
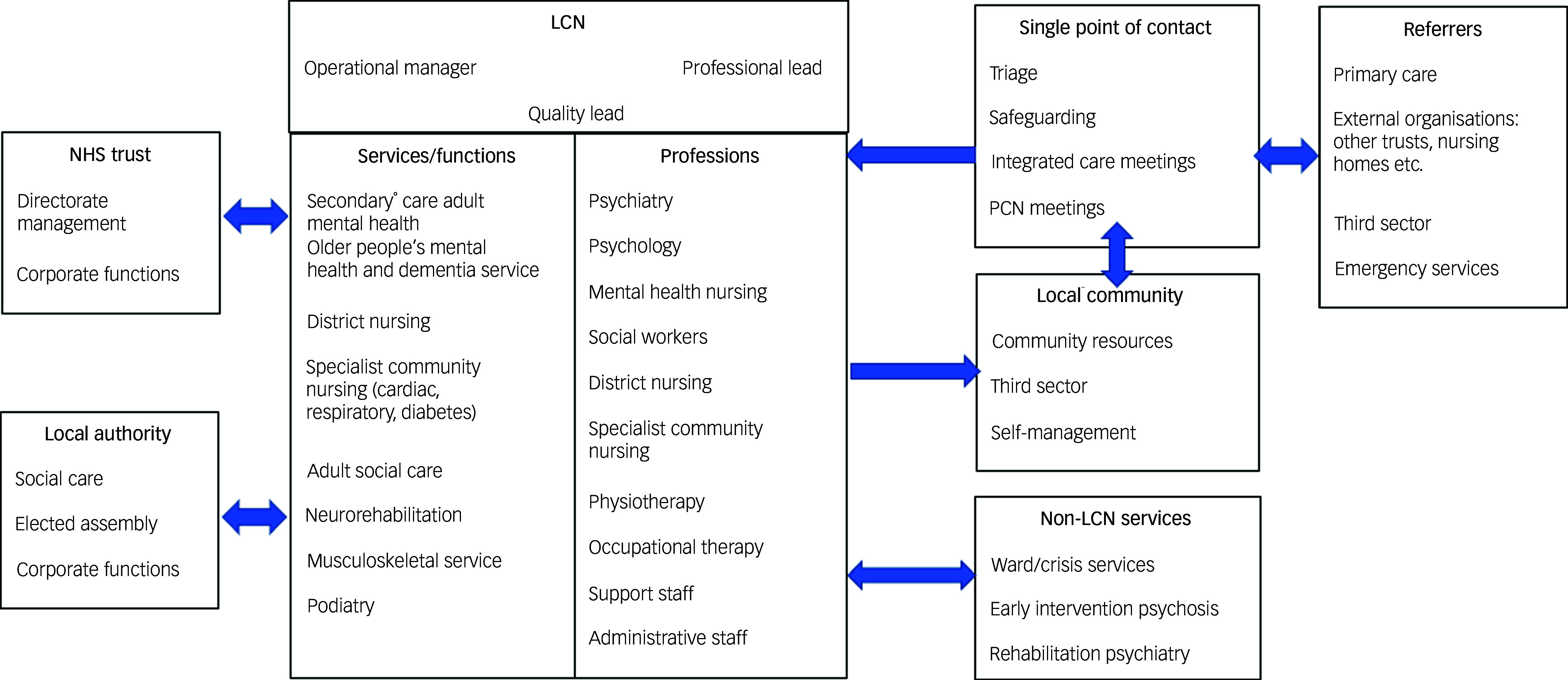
The local care network (LCN), the new integrated team in the micro group. This maps onto care provided by a corresponding general practice primary care network (PCN). The LCN offers a considerably wider range of services and professionals than a typical community mental health team and has fewer interfaces than many such services. In this model, secondary mental health includes general psychosis and non-psychosis care, with the exception of early intervention and rehabilitation psychosis services, which remained separate. The LCN has a single management team, meaning that there are no internal referrals across services within it. There is a matrix management structure in which the LCN operational manager and quality lead may be from any professional group, but each profession has a professional lead for development and training. Most referrals come via a single point of contact that will take all mental health, community physical health- and social care referrals within the borough. Note that in-patient and crisis services also sit outside the LCNs and work across the three LCNs.

The study took place in two London ICSs. The micro group (*n* = 14) was from a borough in south London, where secondary mental healthcare and community physical healthcare services are provided by a single NHS foundation trust, and social care by the coterminous local authority. This community physical healthcare did not include primary care services delivered by general practitioners, but it did incorporate a range of services including physiotherapy, occupational therapy, district nursing and specialist community nursing (e.g. cardiac rehabilitation). Figure 3 gives a more detailed overview. Of note, the NHS trust did not attempt to include all potential or relevant services in this first iteration of a greater integrated service. For example, children’s, addiction and intellectual disability services were not included, although their patient populations clearly have much to gain from such models. It was the organisational aspiration to grow the number of services in local care networks (LCNs) if they proved successful.

Purposive sampling was used to try to ensure a representative range of professional backgrounds to match those delivery and leading services, as well as appropriate coverage in terms of gender, age and ethnicity.

The meso and macro groups came from a north London ICS, with the former (*n* = 14) constituting members of a single NHS trust board and its senior leadership team; this organisation provided both secondary care mental health and community physical health services. However, the latter physical health services were not as extensive in scope as those provided in the first NHS organisation from which the micro group was derived, were limited to one of its three London boroughs of coverage of mental health services and were not integrated into the secondary mental health team in the same manner. The macro group comprised members of the ICB (*n* = 5) to which the meso group worked. See Table 2 for group member characteristics, although some aspects have been anonymised.

**Table 2:** Characteristics of participants in the groups micro, meso and macro

Identifier	Professional group	Title/level	Years of service	Gender	Age (years)	Ethnicity
Group 1 – the micro group						
P1	Social worker	Service manager	15–20	F	45–50	White British
P2	Non-qualified	Quality Improvement manager	5–10	F	30–35	Asian British
P3	Nursing	Psychiatric ward manager	5–10	F	30–35	White British
P4	Social worker	Social worker	<5	F	25–30	White British
P5	Psychology	Consultant psychologist	25–30	M	50–55	White other
P6	Social worker	Social worker	25–30	F	55–60	Asian British
P7	Nursing	Community team manager	>30	M	60–65	White British
P8	Medical	Junior doctor	0–5	M	30–35	Asian American
P9	Social worker	Social worker	5–10	F	25–30	White British
P10	Medical	Consultant psychiatrist	20–25	M	50–55	Asian British
P11	Allied health professional	Physiotherapist	10–15	F	35–40	White British
P12	Nursing	District nurse	15–20	F	40–45	Black British
P13	Occupational therapist	Professional lead	15–20	F	35–40	White British
P14	Nursing	Nursing manager	>30	F	55–50	White British
Group 2 – the meso group						
P1		Chief executive	>30	F	>60	White British
P2		Associate director	>30	F	50–55	White British
P3	Medical	Deputy medical director	>30	F	50–55	White British
P4	Nursing	Clinical director	25–30	F	45–50	White Irish
P5		Associate director	25–30	F	50–55	White British
P6	Medical	Chief operating officer	20–25	M	40–45	White British
P7	Pharmacist	Non-executive director	>30	M	>60	White British
P8	Medical	Non-executive director	>30	M	55–60	White British
P9	Medical	Chief clinical information officer	>30	M	55–60	White British
P10	Medical	Director of research and development	20–25	F	45–50	White other
P11	Psychology	Clinical director	>30	F	55–60	White British
P12		Chief finance officer	>30	M	50–55	White British
P13		Chief people officer	>30	F	50–55	Asian British
P14	Nursing	Chief nurse	>30	F	55–60	White British
Group 3 – the macro group						
P1	Medical	Chief medical officer	>30	F	50–55	White British
P2		Chief executive	>30	M	50–55	White British
P3	Medical	Chief medical officer primary care	>30	F	50–55	White British
P4	Medical	Chief medical officer acute hospital care	>30	M	50–55	White British
P5	Medical	Chair	>30	F	55–60	White British

In terms of moves towards integration, in the micro group this was provided by the local trust borough directorate management team, under the auspices of the trust executive and board. They had been given a remit of the services that were to be integrated (those in Fig. 3), but the granular detail of how this was to be operationalised was left to them to determine, in consultation with local staff. For the meso and macro groups, at their more senior level these were more impacted by legislative change, notably the 2022 Health and Care Act. Of note, the 2025 NHS 10-Year Plan^6^ had not been announced at the time of data collection. Both the ICB and trust had integration leads, drawn from their own staff, to determine the process and overarching outcomes; however, like the micro group, the detail of how this would occur in detail in individual teams was left to local service management to enact with their staff, under consultation and guidance from the trust executive.

The choice of two regions within London was ultimately a pragmatic one: the lead author’s roles and NHS organisations changed during the COVID-19 pandemic. This is discussed further in Limitations, below, as an issue that impacts interpretation of the findings.

### Procedure

The study received approval from the Southwest Cornwall and Plymouth Research Ethics Committee (REC reference no. 19/SW.0131).

Data were collected through individual semi-structured interviews with the lead author (D.K.T.), using stem questions based on our earlier work^30^ (see Appendix available at https://doi.org/10.1192/bjo.2025.10841); all were recorded. The interviews occurred in late 2019 and late 2022/early 2023, each lasting 30–60 min. By the end of the final interview in each group, it was judged that data saturation had reasonably been reached.^31^ Transcription was completed by a single coder, D.K.T. The impact of D.K.T.’s roles in the organisations evaluated is discussed in Limitations, below.

Thematic analysis was considered optimal as an inductive exploratory process to create a new theory from emerging data.^32^ It afforded flexibility in terms of allowing both an inductive and a deductive approach, and could be applied across a wide range of recorded interviews. Mindful of the inherent subjectivity of qualitative work, and risk of biases therein, including that the lead researcher (D.K.T.) worked for the organisation and borough under evaluation, Henwood and Pidgeon’s concept of ‘theoretical agnosticism’ was adopted,^33^ and D.K.T.’s experiences of the assessments, and the emergent themes, were explored in academic supervision with the two senior authors of the paper (K.H. and S.S.S.).

Data were first coded, by D.K.T., according to the overarching and main themes in Rogers’ Diffusion of Innovations framework. Subthemes were identified from the data based on a careful reading of the transcripts. In the analysis process several additional high-level themes, not part of Rogers’ model but which provided additional insight into the process of integration, were identified.

Nvivo 13 for Windows (QSR International Pty Ltd, Doncaster, Victoria, Australia; see https://lumivero.com/products/nvivo) was used in the coding and deriving of themes.

## Results

Overall, there was a noted need to change the service provision model from the status quo, and few were happy with the contemporary health- and social care system prior to any move to greater care integration. Greater integration was perceived to offer many potential gains for patients and carers alike, especially those with complex and long-term conditions, and as a novel learning environment for staff. However, there were concerns that this new model of working with others and in new ways introduced the contrary risk of professionals deskilling and losing expertise in an inherently more generic model. Early integration experiences were showing mixed results: there were gains via improvements in patient experience and ‘flow’, but also some increases in bureaucracy and ‘double-running’ of systems. Policies, both national and local, were seen as unduly optimistic and not capturing practical challenges. Despite considerations on the need to engage staff and tap into cultural factors, there was felt to be a general dearth of this in practice.

The coding structure, together with illustrative quotations, is shown in Table 1. Themes and subthemes arose inductively from the data and are an extension of Rogers’ framework.

Participant quotes are identified by a nomenclature that captures the group but not the participant number, to help maintain respondent anonymity: for example, G1L is group 1 (the micro group) but cannot be linked with any particular participant in that group.

### Prior conditions

Pre-existing conditions – the contemporary NHS landscape just prior to any move to more integrated care – were generally considered suboptimal, including care provided, patient experience, workforce and its workload: ‘the general view of the NHS is that it’s a disaster’ (G3PC). It was thus deemed ripe for change by most. The micro group emphasised on-the-ground inefficiencies that needed to change, such as complex referral systems: ‘not logical … not good patient experience … inefficient because you then have to have two, three appointments instead of the one’ (G1PL).

The meso and macro groups highlighted siloed ‘sovereign organisations … building strategies by themselves … [in] a system where they’re struggling already’ (G3PB), leading ‘to a fragmentation of care and patient experience’ (G3PA). The historic split with social care was noted, where ‘that’s a social work job, and that’s a mental health job … missing professionals in each side of it’ (G2PD).

It was seen as appropriate to change to a ‘joined-up set of public services that are looking after holistically, the individual’ (G3PA), an ‘enabler of a good society’ (G3PB) with the ‘benefit of best outcomes, reduce the … inequalities in both public health, the access to health and care services and … outcomes’ (G3PA). It was also considered that ‘there isn’t enough money’ (G1PC) to continue with the existing models, although such perspectives commonly elicited some scepticism of political drivers for change being expediency rather than better care.

### Innovation characteristics

This explores putative gains of the innovation over the prior conditions. Participants felt that integration offered gains in patient and staff experience, creating a learning environment and facilitated system efficiencies. However, there were concerns that professionals might deskill, there was little opportunity to trial and learn and that measuring success would be difficult.

Care quality was anticipated to improve for ‘people with long-term conditions … particularly for older people’ (G3PC), including joining up with social care for ‘things like housing and employment’ (G2PD). The model was seen as bringing together professionals from historically disparate elements to wrap care around people, offering a system inherently ‘easier to understand’ (G1PD): ‘if … you just call one person, not four, it could be a game changer’ (GP4). It offered opportunities to reduce local health inequalities in marginalised groups.

Workflow was predicted to become smoother, with reduced bureaucracy between services, especially primary care. There were mixed thoughts on what that might mean for workload. Early intervention and timely advice might prevent unnecessary referrals, and a trusted colleague might complete several tasks on a single visit, so a patient did not have to ‘tell their story over and over again’ (G1PD) to multiple professionals. However, there were concerns of increased referrals of ‘lower-intensity’ patients overwhelming the system.

There was a disconnect between the micro and leadership groups in terms of learning opportunities. Most recognised opportunities for being a ‘place people might want new skills’ (G1PM), an ‘extraordinary incubator … of good ideas’ (G3PE) and ‘learning from other people … to talk to … other professions’ (G1PJ). This included through novel posts such as ‘mental health additional roles reimbursement scheme’ (MHARRS) workers and new tasks. However, many in the micro group worried that they would deskill in a more generic environment, and that the nature of work would change to cover multiple erstwhile separate posts.

All agreed that systems needed to be efficient with money, but for the micro group this led to some conflict with their professional values if money was driving change: ‘if we had the money … and the people we needed that we wouldn’t do this. But it’s a way of coping’ (G1PH). Many across all groups foresaw that back-office functions such as information technology and human resources might be most effectively managed at a larger scale, although countering this was some concern that governance issues had not been fully resolved.

### Stage of innovation

In general, the micro group had little awareness of policy or legislative drivers, and a couple of participants argued that this was not necessary: ‘on the ground, they may not be that preoccupied, thinking about that deep vision for the NHS’ (G1PM). The leadership groups largely concurred, caveating that ‘the idea to integrate things better is would be strongly familiar to them’ (G2PN). Nevertheless, there was unanimity on the subsequent theme of ‘persuasion’, with integration offering superiority to the current model.

The subtheme of ‘relative position’ arose inductively, with all groups feeling nationally advanced insofar as they thought their level or degree of service integration was greater than that of most comparator organisations, albeit there was limited empirical evidence provided to support this hypothesis. This was commonly without evidence, although the micro group correctly noted that their integration with social care was novel, and several in the meso and macro groups identified the shared patient database ‘WISC’ as advanced. Advancement attracted negativity insofar as ‘people don’t always like being a pilot’ (G1PA), and ‘there’s a lot of cynicism … [about] being early adopters’ (G2PI), because this meant advancing without guidance on effective change. Rogers’ stage of ‘decision to adopt or reject’ did not apply insofar as integrated care is nationally mandated. However, this produced the problem of integration feeling enforced ‘even if it’s for the better’ (G1PK).

In the ‘implementation’ theme, participants described actual working experiences in integrating services, allowing reality testing of anticipated changes from the prior conditions. Positively, hoped-for gains were seen in terms of improved patient experience, care quality, workflow, reduced bureaucracy, learning from others and enjoyment of a wider professional network:

‘it’s quite nice … continual conversation about changes … new services, ways of working … makes people more … efficient … personal relationship[s] with … different professionals that work in different ways’ (G1PD).

However, greater workloads were also emerging: ‘we really face being overwhelmed with demand’ (G1PH).

Interestingly, reductions in back-office inefficiencies were not occurring; indeed, there were significant concerns about duplication of effort, from increased meetings to double entries on record systems. Included was an initial ‘double-running’ of parallel systems and seemed likely to settle. However, there were intractable problems such as different email and electronic patient record systems and contractual variation between health- and social care staff. The micro group felt that, in terms of practical issues, ‘there has not been enough attention paid by management. Staff keep saying this and it feels as if it’s not being listened to’ (G1PE).

The leadership groups felt that financial cooperation was improving, with the caveat from several that past unhelpful behaviours of partner organisations instilled an inherent caution. More problematic was an emerging schism with the local authorities, who were generally perceived to not be engaging fully and quite variable in approach: ‘some are really pro it and some are really against [integration]’ (G2PC), influenced by the political make-up of their councils.

### Adopters’ characteristics

New ways of working and team professional make-up challenged many participants in the micro group’s sense of identity: ‘everyone’s in a minority’ (G1PA) and ‘no one [is] belonging’ (G1PM). This had particular resonance for social care staff, who commonly felt even more minoritised:

‘We’re partners but we’re doing it because of NHS drivers. But to be honest we always feel that. Social care always feels in the shadow to health … I think social care has a bit of a chip on its shoulder when it comes to the NHS’ ( G1PD).

Healthcare participants also noted differences from social care colleagues: ‘I don’t understand what you’re saying … it feels a different country … the way that they do things … it’s going to be quite some time … before a shared identity emerges’ (G1PM). Team lanyards were altered, including a removal of the NHS logo to emphasise merging with social care, but this provoked an unexpectedly strong backlash: ‘symbols of a team and its identity are not just practical things … they are important to the staff’ (G2PA). The general sense from frontline participants was that their leadership team ‘struggled to cope with … if they ever really got it [the identity challenge]’ (G1PG). However, there was nuance, with some comments such as ‘our strong professional identities … it doesn’t help patient care … it overshadows people’s needs, which are complex and bigger than any profession’ (G1PB).

Much policy documentation on integration noted the opportunities, or necessities, of working in different ways, including novel roles, such as physician associates, and new responsibilities, such as non-medical prescribers. The meso and macro groups were largely positive about ‘get[ting] all professionals working … to the top of their licence’ (G2PH) – a managerial phrase invoked by several micro group participants as ‘infuriating’ (G1PA). Leaders spoke of developmental opportunities that were ‘really exciting … no longer labelled “just” a nurse or an OT or whatever … a real opportunity to grow to something new’ (G2PC) that ‘may help with retention … barriers to entry … there should be a win-win in there if we can get it right’ (G2PH).

The micro group positively noted ‘opportunities to push our envelope’ (G1PM) ‘on the edges of our job role … to deepen our professional knowledge’ (G1PL), appreciating ‘nice teams to work in … with the bigger group of professionals, learning from them of thinking in different ways’ (G1PK). Nevertheless, they were largely sceptical about novel roles or changes to their core roles, typically feeling that the former were ill-defined and that the latter were not what they trained for or wished to do. These were generally perceived as ways to save money.

Only the meso and micro groups discussed leadership attributes, deemed essential to ‘unlocking some of the barriers to more integrated working’ (G2PA) and to ‘set the tone … in its conversations’ (G3PD), even if necessary ‘compassionate leadership, empathetic leadership’ (G3PA) was challenging in busy systems. There were concerns: ‘we don’t give enough time to thinking about our leaders and leadership’ (G3PB), leading to ‘tension … you see politics’ (G2PA).

Culture was seen across all groups as ‘the big one … [but] so difficult’ (G2PD), ‘the whole culture piece hasn’t really been thought of in ICS … you know, what are we here for’ (G2PH), in part as it ‘takes time to do’ (G3PB), ‘a 5-, 10-year job … a hearts and minds of all elements of the system’ (G3PA). One of the most profound group differences occurred in the cultural interface with social care. The micro group now had mental health, physical health and social care staff in single new teams. Participants noted the variation between healthcare’s ‘deficits-based model’ (‘what’s wrong with you?’) and social care’s complementary ‘strengths-based model’ (‘what are you good at?’). Several said that while they had been aware of the other philosophy, the impact of seeing it practised directly by new colleagues was profound, with staff starting to model others’ behaviour. The gains were bidirectional and the richness in their combination: ‘more of the client-centred strengths-based work’ (G1PD), ‘we [healthcare] give something back [to social care] in terms of our evidence-based approach’ (G1PF).

It was a professional shift in ‘ways of seeing’ (G1PD), that ‘isn’t taught, but you copy others when you see what they’re doing is right and works’ (G1PA). Interestingly, this was unexpected and organic – the aim of more integrated teams being proximity and practicalities – and further that the leadership did not seem certain of how to maximise this: ‘I don’t think it occurred to them, or if it did, they didn’t say anything’ (G1PE).

### Communication behaviour

Micro group participants expressed recurring frustration at ‘not [being] listened to’ (G1PH) in the design of local integration, sensing that ‘it is being done without us’ (G1PC). The outcome was that, while participants agreed with the ambitions of change, communication breakdown ‘didn’t go down well’ (G1PD), hindering engagement: ‘it makes me angry’ (G1PC). Participants noted that the changes were also hard for the leadership team, but their limited responses did not help. The leadership team had arranged for a question-and-answer website, an email box for queries and several large ‘town hall listening events’, but these were perceived as ‘corporate bullshit’ (G1PF), with no clear linking to any change or learning from feedback.

The leadership groups echoed that ‘you can’t over-communicate’ (G2PA), because if you do not, ‘you can have the most brilliant strategy if you do not take people with you’ (G3PB). However, they also agreed that communication remained inadequate, ‘typical of NHS reorganisations … top down … No one talks to the frontline staff’ (G2PH), telegraphing that ‘this is the model and this is what we’re doing’ (G2PF). The meso and micro groups also noted a failure to catch the successes of the programme, ‘celebrating the things … making a difference and the changes … the messaging isn’t strong’ (G2PD).

### The surrounding social system

Rogers’ model describes innovations occurring in a wider social system, with different strata or ‘units’ of diffusion. In this setting, units might be considered clinical teams, NHS trusts and the local authorities, the ICB and the London region and the national picture/government. Accordingly, most felt that relationships worked well within their unit, with challenges moving across them; there was a general tendency to put more blame on the other levels of unit for any such friction.

Frontline teams were heavily impacted by reorganisation, with removal of erstwhile, often longstanding, colleagues and insertion of new ones from different professional groups. Interestingly, after getting over any initial shock, many were positive about ‘meet[ing] with people in teams that I would otherwise not met’ (G1PI) who were ‘intimately involved in our service users lives’ (G1PM).

There were interesting parallels between the three groups where they identified a ‘parent–child relationship’ (G1PE) tension with the level above them. The micro group often felt ‘a little person … [to those managers who] paint with corporate speak’ (G1PA); in their turn, the meso group expressed similar thoughts on their relationship to the ICB, and the macro ICB group regarding NHS London/England. There was some concern from the meso group that mental health was less prioritised by the ICB and ‘at the receiving end of the perceived wisdom of the acute trusts’ (G2PA).

The most challenging unit interface for the meso and macro groups was with the local authorities, where profound and seemingly insurmountable differences persisted. Structurally there was a ‘fundamental tension between local democratic element of the [eight] local authorities and councils’ (G3PA), each of whom was felt to be too inwardly focused on their own residents without considering wider systems, and too beholden to local councillors of very different political persuasions:

‘you get out in [Conservative-controlled borough] and it’s we’re gonna hate the EU and we don’t want a nanny state … into [Labour-controlled borough] … another world … you’re stuck between these very, very, very stark political views of councillors as much as their residents’ (G3PC).

## Discussion

The population, workforce and financial drivers are unambiguous on the need for more integrated care internationally; legislation and policy in the UK have tracked this with mandated moves to ICSs, reinforced through the most recent 2025 NHS 10-Year Plan.^6^ This work set out to determine the impact of policy and early practice towards more integrated care across three staffing levels in London. The qualitative data show that participants at different organisational levels support the principles of these moves, and found emerging benefits in terms of patient experience and care, and training and learning opportunities. However, failure to capture the issues that matter to them, not least the practical challenges of change and early evidence of increased workloads, to capitalise on the emerging anecdotal gains and problems, and to grasp the profound cultural opportunities and challenges of integration with social care, are hindering effective diffusion of this necessary innovation. Several key findings emerged.

First, while legislation and policy documents have efficiently captured the need for change and putative gains, they are largely optimistic in their objectives and tone, failing to anticipate or describe challenges. The long-term plan^4^ that underpins much of the change in the UK has sections on how to ‘empower people’ and ‘improve population health … clinical efficiency and safety’ that avoid mention of downsides. Similarly, the NHS Workforce Plan^34^ describes a workforce helped by ‘digital and technological innovations’, yet without describing inefficiencies or duplication. They do not address ‘cuts’, deskilling or unwished-for new forms of work. None speak to the impact on professional value sets or suggest the need for leadership engagement and discussion.

Participant reports support the emergence of both quality and efficiency gains and feared workload increases. The care provided was deemed to be showing improvements with fewer barriers, and good cross-working with primary care. The experience reported on increasing workload aligning with French data on integration of services, also post-pandemic, that showed a considerable rise in workload, greater than predicted, that professionals had difficulty absorbing in a novel care coordination role.^11^

This reflects a second finding that, without clear guidance, little monitoring and evaluation was being undertaken, hindering learning and improvement. Kozlowska et al’s review of integrating systems internationally noted that change is facilitated by sharing learning,^35^ and here, participants suggested sensible mechanisms for measuring change in new models, but little was occurring in practice. More recently, there have been some proposals to evaluate ICSs, but such an occurrence is after their instigation and local approaches remain largely absent. In a contemporaneous era of increasing focus on healthcare productivity,^36^ this feels a significant miss.

Third, early experiences with information technology and other proposed back-office efficiencies were often counterproductive. Evidence of any back-office financial savings were limited by system novelty, and concerns around organisational protectionism persist.

Fourth, staff identity, effective leadership through change and organisational culture were considered critical factors in effecting successful integration. However, these were felt to be largely forgotten or ignored, hindering implementation. There was a desire from the meso and macro groups for a more ‘flexible’ workforce with new roles and opportunities that aligned with policy,^4,34^ although such documents risk appearing largely aspirational in approach, with less detail on approaches to managing change.^34^

However, such approaches fail to respond to the micro group’s anxiety about being professionally undermined and deskilled. Interestingly, this professional anxiety did not appear to be being borne out in practice in our data, with more enthusiasm about experiences of learning and working with others, fitting with Hovlin et al’s finding of positivity from ‘different viewpoints provided by the different professions’.^7^ Nevertheless, it demonstrates a potential recurring communication gap: managerial and policy statements of ‘working to the top of your licence’ and ‘upskilling’ produced real ire and compounded a sense of not being heard. These findings fit with three of the six recommendations from Kozlowska et al’s early review of integrating systems^35^: engage stakeholders, address the unexpected consequences of change and train the workforce for collaborative working.

The importance of a healthy organisational culture and leadership is not a new idea. The Nuffield Trust has noted how ‘Successful integration often comes down to good relationships at a local level, leadership and an amenable culture’.^37^ However, what is novel in these data is how profoundly these elements have affected integrated care in practice, that they could be as deeply positive as negative and how there was failure to plan for this across various levels. Culture and identity are almost entirely unmentioned in the major UK policy and legislative pieces.

In terms of leadership in integrated healthcare, there are governmental and expert-led documents on this topic^38–40^ that speak of the determination and goodwill of staff and leaders to improve care, but of systems that are siloed, lack accountability and do not always encourage or reward collaborative behaviour. Its recommendations recognise the need for good leadership and suggest deliverable targets for these, such as data on demographics and equity of senior leadership positions, and numbers in leadership training. These typically distil what are considered the key values of ‘the heart, head and hands of leadership’ in healthcare, such as ‘we are compassionate; we are curious; we are collaborative’, although they often lack detailed, actionable points. All three groups in our data emphasised the need for good, accountable, inclusive leaders but all respondents said, in different ways, that stretched busy systems were not actively supporting this in terms of delivering more integrated care.

Participant perceptions of a lack of good communication threads much of these mismatches. Lewis’ work on ‘what can we learn from a decade of national pilot programmes?’ in the UK found ‘there was little stable or shared’ communication.^41^ In this current work, there were perceptions of a widespread failure to demonstrate hearing of concerns, as well as positive outcomes through clear communication plans showing what has subsequently changed and been learned. Linked to this was a failure to communicate that some of the frontline staff anxieties were not being borne out: care was proving better for many, with more fluid cross-working; learning seemed to be trumping deskilling; and cultural gains were outweighing new team anxieties. An outcome of the failure to do this has been a strain on relationships at multiple levels, with a negative impact on trust and a hindering of integration implementation.

The cultural and practice benefits from better integration with social care have not been fully realised. Rather than proximity, the real gains are changes in how staff work and think about care.^42^ The ‘deficits (but evidence)-based’ model of healthcare and the ‘strengths-based’ model of social care are highly complementary. Uittenbroek et al’s work on Dutch social workers and district nurses in a new ‘case manager’ integrated team identified some of this, although the findings focus on a compensation of the ‘other’s gaps’ rather than on a cultural shift per se.^9^ Regarding the higher-level ICS aspects, participants from the meso and macro groups perceived that integration was fundamentally hindered by the variable make-up, priorities and engagement of local authorities of differing political hues.

A UK government-commissioned piece exploring integration noted that, if integrated care is to succeed, social care needs to be a priority for investment and workforce.^8^ However, this primarily reinforced the critical and still unresolved issue of the funding model for social care. Policy is in place, in terms of the Health and Social Care Integration White Paper,^43^ which also speaks to leadership and culture. However, it is presented largely on an aspirational basis of principles that ‘good’ and ‘effective’ leadership are necessary, rather than detailing what that might look like in practice. The King’s Fund has noted the ‘differences in language, spending power, culture and leadership style’ between health- and social care, and called for a strong social care representation in ICSs.^44^ That report noted the cultural strengths that social care brings, and how social care leaders could be ‘healthy disrupters’ at a senior leadership and systems’ level.

Our data affirm the challenges, but enormous opportunities, that are brought about from having social care as a partner in integrating systems. At its best, it was an area where frontline joint working and cooperation led to a stimulating flourishing of ideas, complementary styles and cross-learning. At more senior levels, it was typically borne out through dissent and a fundamental sense of two different and fundamentally divided philosophies with a poor history of working together.

### Limitations

The lead author worked at senior levels within the systems evaluated, which may have impacted participants’ sense of ability to feed back critically. The participants in the more senior leadership group had less ethnic diversity than the micro group, albeit this is a noted challenge across the NHS more generally.^45^

Data collection was interrupted by the Covid-19 pandemic, extending the time for collection, and interviews in the meso and macro groups were inevitably impacted by their experiences during this time. Notably, the 2022 Health and Care Act had been enacted in law at the time of the second and third groups’ interviews, and thus they were working in a landscape of more formal structures and greater general awareness of these. It is possible that this impacted participants’ feedback, not least because the legislative changes were most likely to impact and be absorbed by those two latter more senior leadership groups.

It was not originally intended to collect data from two different ICSs; this was an outcome of the lead author changing roles and NHS trusts during the pandemic. When the micro group speak of managers and leaders, they are talking of individuals not subsequently interviewed in this study; equally, when the meso and macro groups speak of their frontline clinicians, it is of a different cohort to this micro group. While there were many commonalities across the micro, and the meso and macro groups, this is a challenge to the validity of the data.

A significant number of the results concerned social care. While the micro group contained participants from social care backgrounds, the meso and macro groups did not. It would have been helpful to have the perspectives of senior social care leaders from within the ICSs. While the macro group included ICB leaders who also worked clinically in primary care and large acute physical health hospitals, they spoke primarily in their ICB role. It is not clear how generalisable the findings here are to either primary care or acute hospital care, or their parallel moves to more integration in the community.

The true measure of the impact of more integrated care will be in its effectiveness to patients and carers alike. This work explicitly did not focus on those groups.

Finally, legislative changes that followed the completion of the work mean that NHS England will be absorbed into the Department of Health and Social care over the coming 2 years.^25^ Furthermore, in 2025 the UK Government published a new 10-Year Health Plan for the NHS^6^ that focuses on three strategic shifts, moving from hospital to community, sickness to prevention and analogue to digital.^6^ The exact impact of these upon integrated care, and the findings in this paper, are as yet unknown.

### Reflexivity statement of the lead author

I worked as the clinical director of the micro group and was the chief medical officer of the meso group that worked to the macro group. As noted in Limitations, above, it seems reasonable to hypothesise that this seniority might have impacted participant feedback; the research aim of attempting to try to understand, evaluate and improve services with staff, while positive in intent, might equally have skewed what was said. Following on this theme, I was the sole coder of the data and it is possible, and indeed likely, that my own perceptions and experiences impacted how I did this, even with a clear intent to be as ‘neutral’ and faithful to the core data as possible. Supervision with the two senior authors of the paper (K.H. and S.S.S.), neither of whom had ever worked in the services evaluated, helped with this fidelity.

I have been profoundly impacted by undertaking this work, primarily through my recognition that management attempts to engage staff are seldom as adequate, deep or authentic as staff would wish, and that they tend to speak to national drivers and trends rather than address issues that are at the forefront of staffs’ mind. Furthermore, it has been a deep learning experience on the putative gains of cultural change, yet how inadequately we focus upon this, or the human factors that lead to success and failure in processes of change.

### Implications

Our findings are mixed, in that participants largely support the drivers for integration but their concerns, and means for measuring change, have largely been ignored, limiting learning and integration optimisation. Culture, leadership and the benefits from closer working with social care all need greater emphasis.

A key problem is that models vary and there is no agreed way to measure integration, change or success.^30,35^ NHS England has put forward its ‘Core20PLUS5’ agenda^46^ to reduce healthcare inequalities locally and nationally. The results in this manuscript offer various alternative areas that might be explored.

There is a wide literature on effective leadership directly exploring culture and relationships.^39^ A curious, questioning integrating team, service or organisation might look to actively identify and resolve or mitigate problems, identify good practices, foster relationships and leaders who were supporting these and disseminate learning at all levels.

## Supporting information

Tracy et al. supplementary material Tracy et al. supplementary material

## Data Availability

Primary qualitative interviews are available upon written request to the lead author. The lead author maintains accurate records of the data, and these can be supplied upon reasonable demand.

## Footnotes

a. The four UK devolved nations each have their own NHS regulatory body and have had varying legislation passed that impacts each differently, although the principles of more integrated care are common to all. This paper focuses on England and NHS England in terms of policy and service delivery.

## Supplementary material

The supplementary material is available online at https://doi.org/10.1192/bjo.2025.10841
