# Supplementary material for: Integrated care in practice: lessons from three tiers of healthcare provider and commissioner staff in two London Integrated Care Systems
Source: BJPsych Open. 2025 Sep 15;11(5):e214. doi: 10.1192/bjo.2025.10841 (PMC12451555; doi:10.1192/bjo.2025.10841)
Supplement: Tracy et al. supplementary material [file S2056472425108417sup001.docx]

| **Group 1 (micro) interview questions** |
| --- |
| 1. What has been your understanding of the drivers or reasons to move to greater ‘integrated care’ in Bexley? (Potential sub-prompts: population and/or workforce drivers; different models of care; service pressures such as infrastructure/estates, IT, finances) 2. What is your understanding of how this fits into a wider national move or model of integration, and where do you think Bexley is in relation to this? (Potential sub-prompts: awareness of national/NHS policies, changes?) 3. What do you see as the potential benefits of a new integrated model, and integrated models more generally? (Potential sub-prompts: for patients/carers, for staff, for services & organisations) 4. What do you see as the potential challenges or downsides of our new integrated model, and integrated models more generally? (Potential sub-prompts: for patients/carers, for staff, for services & organisations) 5. How might we know or measure if this integrated model, or integrated care more generally, was working, or not, in say one to two years’ time? 6. How involved and engaged have you felt in the local change programme? 7. What might local management or staff, or national models, do differently based upon your experiences? |
| **Group 2 (meso) interview questions** |
| 1. What is your personal sense of the main drivers and potential gains of more integrated care and ICSs, both nationally and within our NW London sector? [possible prompts on care quality, finances, efficiency and back-office functions] 2. How well placed do you see [names Trust] and the London B ICS in the London region and nationally, and what are our local facilitators and barriers? 3. Are there more natural ‘winners’ or ‘losers’ in the ICS, between: organisations (primary, community, acute, mental health care); teams within [names Trust] (e.g. CAMHS, older persons); and patient or condition types? 4. What is the role of the local authority in integrated care and the ICS, and how are we placed in that regard in our London B ICS? 5. What is your sense of frontline staff understanding, perceptions, and involvement in the national drivers, the ICS and any Trust- or team-level changes? 6. What are the roles and influence of leaders, relationships, and culture upon integrated care at a team and systems level? 7. Several core themes emerged from earlier research on frontline staff in a different NHS Trust. Each had positive and negative components, and I wonder what your reflections upon these and local frontline staff are:    1. Professional identities [possible prompts: tasks, workloads, morale, responsibilities]    2. Learning opportunities [possible prompts: upskilling and deskilling]    3. Practical challenges [possible prompts: team bases, desks, car-parking, IT] 8. Looking back, what learning can we take from work so far? Looking forward, where do you see the ICS say five years from now, and how might we measure successes or failures? |
| **Group 3 (macro) interview questions** |
| 1. What’s your personal sense of the main national and ICS drivers & barriers for more integrated care?   [possible prompts: better care, more efficient, finances, workforce, IT/data, estates]   1. How well placed are we within our London B ICS; what are the local facilitators and barriers, and who (patients, staff, organisations) does it help most or least? [Prompt re primary care, community health if not mentioned; how ‘equal’ are partners?] 2. Where do culture & leadership come into (or inhibit) delivering this, beyond ICB plans, structures and so forth? 3. How well are we doing/can do engaging patients, carers, third sector, especially on a preventative public health and reducing inequalities agenda? How does ‘firefighting’ crises impede this? 4. Where is social care in the ICB? How well are we doing with social care? 5. How aware and engaged do you think front line staff are in the ICB, ICS, integrated care, and how much does it ‘matter’? 6. What are your thoughts on challenges of professional and organisational identity and deskilling as organisations and roles ‘merge’ and change. 7. Where are the opportunities and challenges re workforce, recruitment & retention in the new model? 8. Where do you see the ICS five years from now, in broad terms of care delivery? How would we know/measure if it was working or not? |
